# Angiotensin II inhibitor facilitates epidermal wound regeneration in diabetic mice

**DOI:** 10.3389/fphys.2015.00170

**Published:** 2015-06-09

**Authors:** Maria Kamber, Vasileios Papalazarou, Georgia Rouni, Evagelia Papageorgopoulou, Apostolos Papalois, Vassiliki Kostourou

**Affiliations:** ^1^Biomedical Sciences Research Centre “Alexander Fleming,”Athens, Greece; ^2^Experimental Research Center ELPEN PharmaceuticalsAthens, Greece

**Keywords:** wound healing, angiotensin II, endothelial disfunction, tissue regeneration, diabetic

## Abstract

Tissue regeneration and wound healing are severely impaired in diabetes and are associated with poor circulation and dysfunctional blood vessels. Angiotensin II inhibitors are anti-hypertensive drugs used in clinical practice to regulate blood pressure and could affect tissue remodeling. We hypothesize that blocking angiotensin II, using Losartan, could facilitate tissue regeneration in diabetic mice. To this end, we established an experimental model of wound healing in streptozotocin-induced diabetic mice. Our data demonstrated that Losartan accelerates wound repair and normalizes wound stromal responses, having a beneficial role in wounds of diabetic individuals. Our findings highlight a potential therapeutic use of Losartan in improving wound repair in diabetic conditions.

## Introduction

Tissue regeneration is required for restoring organ function after injury or during the renewal of tissues. The most common example of tissue regeneration in the adult organism is wound healing. Wound healing is a dynamic process requiring soluble mediators, blood and parenchymal cells, as well as extensive extracellular matrix remodeling (Eming et al., 2014). Wound repair can be divided into at least three overlapping processes: an initial phase that involves a vascular response leading to hemostasis and an inflammatory reaction protecting the organism from pathogen invasion, a second phase that contributes to tissue regeneration and a third phase that comprises a tissue remodeling process (Werner and Grose, 2003). Initially, the damage of the blood vessels during a deep injury triggers platelet coagulation and the development of fibrin clot that temporary seals the wound. Both platelets and infiltrating inflammatory cells release growth factors, cytokines, and proteinases that contribute to the formation of new tissue. The second phase is characterized by extensive cellular activity, which involves proliferation of keratinocytes at the wound edge and migration into the wound where they form a multilayer undifferentiated epidermis. Hyperproliferative and migrating keratinocytes express specific keratins of the basal epidermal layer, which denote a poor level of differentiation, such as keratin 14 and keratin 5 (Alam et al., 2011). Concomitantly, fibroblasts migrate into the wound and differentiate into myofibroblasts contributing to wound contraction and to the deposition of additional matrix, mainly in the form of collagen. The new tissue generated at the injured dermis- called granulation tissue- is rich in inflammatory cells, myofibroblasts and newly formed capillaries. The development of new vasculature at the wound edge is necessary to sustain the newly formed tissue and to support keratinocyte migration, though the secretion of growth factors and extracellular matrix remodeling (Gurtner et al., 2008). At the last phase of would healing, a reduction in cellularity and extensive extracellular matrix remodeling, contribute to differentiation and resolution of keratinocyte layers and the regression of the granulation tissue in the dermis, thus restoring tissue structural integrity and physiologic functionality.

Impaired wound healing responses and chronic ulcers are the hallmarks of diabetes, a metabolic disease that affects many individuals worldwide. According to the WHO the global prevalence of diabetes in adults is 9% for the 2014 and more than 1.5 million deaths are directly attributed to diabetes. Wounds of diabetic mice exhibit prolonged inflammation, microvasculature aberrations that result in irregular circulation and diminished angiogenesis and decreased collagen production (Eming et al., 2007; Seitz et al., 2010). The expression of various growth factors, such as the keratinocyte growth factor, is either reduced, delayed, or absent in the wound tissue of diabetic mice, especially during re-epithelisation and granulation tissue formation (Werner et al., 1994). Similarly, the production of pro-angiogenic growth factors, such as the vascular endothelial growth factor (VEGF) and members of the family of fibroblast growth factors (FGF), which facilitates the wound healing process is greatly decreased or absent (Frank et al., 1995; Xu and Zou, 2009). Consistent with an important role for the microvasculature in repairing wounds of diabetic mice, the topical application of VEGF or *ex vivo* cultured endothelial progenitor cells seems to accelerate the wound repair in diabetic mice (Galiano et al., 2004; Asai et al., 2013).

Evidence suggests that components of the Renin-Angiotensin system, known as the RAS plays an important role in the pathogenesis of wound repair and tissue reconstruction in diabetes (Cooper, 2004). Specifically, angiotensin II, the active biological component of this system, acts through two receptor subtypes AT1 ans AT2 to control systemic blood pressure and blockers of Angiotensin II action are systematically used in clinical practice as anti-hypertensive drugs. Clinical evidence suggests that the use of AT1 inhibitors in diabetic patients reduces the risk of cardiovascular and renal complications (Cooper, 2004; Rask-Madsen and King, 2013). Apart from its function in controlling blood pressure, Angiotensin II regulates cell proliferation, migration, and collagen metabolism (Ren et al., 2013); all processes important for wound healing. Therefore, we hypothesize that inhibition of Angiotensin II could facilitate tissue regeneration in diabetes. To this end, we established a wound healing protocol in streptozotocin-induced diabetic mice and examined the effect of a clinically widely used, competitive AT1 inhibitor, Losartan, Gavras and Salerno (1996) in regulating tissue regeneration.

## Materials and methods

### Experimental mouse model of streptozotocin (STZ)-induced diabetes

Male mice C57BL/6J, 8–10 weeks of age, were fasted prior to STZ (Fisher Scientific) administration for 4 h. A solution of 22.5 mg/ml STZ in sodium citrate buffer was prepared fresh before each injection. Mice were weighted and the appropriate volume of STZ-citrate solution was injected intraperitoneal (i.p.) in each mouse, so that the final dose was 150 mg STZ/Kg mouse. STZ-treated mice were supplied with 10% sucrose in water overnight to protect against sudden hypoglycemia. Glucose levels were measured 2 days after STZ administration and then monitored closely for 2 and 4 weeks, using AlphaTrak glucose meter and strips, specially calibrated for mice (Abbot). Mice became diabetic (blood glucose levels above 400 mg/dl) 2–4 days post STZ injection. Diabetic mice received daily subcutaneaous injections of insulin. At 4-weeks post STZ administration, diabetic and control mice were treated with the angiotensin II inhibitor Lozartan for the 14 days duration of the wound healing experiment. Losartan was obtained as pills from the pharmacy, crushed, dissolved in PBS and administered by oral gavage, to control and diabetic mice, daily, at a concentration of 10 mg/Kg.

### *In vivo* wound healing

Skin wounds of 2 mm diameter (2 wounds per mouse) were generated, using a sterile biopsy punch, in the shaved dorsal back of the different experimental cohorts of 6–8 mice, per condition, 2 and 4 weeks post-induction of diabetes. The *in vivo* wound healing experiment was repeated 3 times. Wounded skin was collected at different time points of the healing process for analysis.

### Hematoxylin-eosin staining and masson-trichrome staining and morphological analysis

Excised wounds were bisected along the arterior-posterior axis of the skin, fixed in 4% PFA, de-hydrated and then embedded in paraffin. Sections of 4 μm thickness from the middle of the wound were stained with hematoxylin and eosin, to examine wound morphology. At day 3, the wound width was quantified as the distance of the gap between the two migrating multilayered epithelial fronts across the wound and expressed as % open wound = [wound width (mm) ^*^100/ initial wound size of 2 mm]. The collagen fibers were visualized using Masson-trichrome staining. At day 7, the granulation area of the wound was measured as the distance of the gap between the blue stained collagen fibers of the two sides of the wounded dermis (mm). Keratinocyte thickness was estimated by measuring the depth of the epithelial multilayer in the middle of the wound. All images were captured from a Nikon Eclipse TE 300 light microscope. Areas of granulation tissue, keratinocyte thickness and open wound were measured using Image J (National Institutes of Health, Bethesda, Md.). All quantifications were performed by two different investigators, in a double-blinded fashion.

### Immunohistochemistry

Formalin-fixed and paraffin-embedded tissue sections were de-paraffinized in xylene, rehydrated through graded alcohols and stained for endothelial cells, α-smooth muscle actin (α-SMA)- positive cells and undifferentiated keratinocytes. Antigen retrieval was performed using Proteinase K solution (10 mg/ml) at 37°C for 15 min. The sections were then blocked in 2% goat serum and 1% BSA solution in PBS and incubated with primary antibodies against endomucine (1:100, Sigma), α-SMA (1:200, Sigma) and Keratin-14 (1:50, Covance), overnight, at 4°C. After washing with PBS, tissue sections were subsequently incubated with anti-rat Alexa Fluor 546 antibody (1:500, Molecular Probes) for 60 min at room temperature and mounted in mounting reagent. DAPI was used to stain cell nuclei. Fluorescence images were acquired using the AxioObserved Z1 Microscope (Carl Zeiss, Germany). Quantification of immunofluorescence was performed in a blinded fashion by measuring the pixel area of the fluorescent intensity for endomucin and α-SMA staining, respectively, by Image J (National Institutes of Health, Bethesda, MD).

### Ethical regulations

All animals were housed at BSRC Al. Fleming animal facilities and all experiments were performed in accordance with the European Legal Framework for the Protection of animals used for scientific purposes (European Directive 86/609/EEC).

### Statistical analyses

The non-parametric Mann-Whitney U rank sum test was performed for the quantitative analysis of wound repair. Values are expressed as mean ± SEM. *P*-values less than 0.05 were considered statistically significant. Statistical analysis was performed with GraphPad Prism, Version 4.0 (GraphPad Software Inc., La Jolla, CA, USA).

## Results

### Establishing the tissue regeneration diabetic mouse model

To study the potential interplay between hypertension and tissue regeneration in diabetic conditions, we combined the extensively used streptozotocin-induced diabetic model (Wu and Huan, 2008) with the *in vivo* tissue regeneration model of skin wound healing (Gurtner et al., 2008; Wood and Eming, 2012; Eming et al., 2014). Streptozotocin (STZ) is a nitrosurea compound derived from *Streptomycin achromogenes* that is toxic for the pancreatic β cells and causes pancreatic dysfunction associated with local immune responses, leading to hypoinsulinemia and hyperglycemia in animals (Rees and Alcolado, 2005). Administration of high doses of STZ (150 mg/Kg) generated diabetic mice with high blood glucose levels (Table 1). Diabetic mice were treated with insulin to avoid adverse effects of hypoinsulinemia and hyperglycemia, including dehydration, severe weight loss, extensive nephropathy, and neuropathy. Under the experimental conditions used, the weight of diabetic mice was similar to control (Table 1), indicating the lack of diabetic complications 4 weeks after STZ injection. To establish the tissue regeneration model, male C57BL/6J mice were split into experimental cohorts (6–8 mice): 4-weeks Diabetic mice, 4 weeks Diabetic mice treated with Losartan (Diabetic and Los), non-diabetic (ND) mice, and non-diabetic mice treated with Losartan (ND and Los). We didn't observe any difference between ND and ND treated with Losartan (Supplementary Figure 1) and therefore we merge the data of these groups to generate the control group. The anti-hypertensive drug, Losartan- an inhibitor of Angiotensin II function- was administrated during the wound healing process. Daily oral administration of Losartan did not alter the blood glucose levels, nor had any effect on the weight of control and diabetic mice (Table 1). We performed skin wounds in the dorsal back of all cohorts, and we assessed the ability of skin to regenerate under the different conditions of the experimental groups.

**Table 1:** **Glucose measurements and weight in different experimental groups**.

**Glucose measurements**				
	**ND**	**ND and Los**	**Diabetic**	**Diabetic and Los**
Before STZ (mg/dl)	145 ± 15 (*n* = 12)	157 ± 40 (*n* = 14)	138 ± 15 (*n* = 13)	169 ± 26 (*n* = 16)
2 weeks after STZ (mg/dl)	176 ± 33 (*n* = 12)	176 ± 20 (*n* = 14)	588 ± 73 (*n* = 13)	580 ± 112 (*n* = 16)
4 weeks after STZ (mg/dl)	187 ± 25 (*n* = 9)	202 ± 46 (*n* = 12)	563 ± 119 (*n* = 9)	558 ± 128 (*n* = 13)
Weight (g)	21.8 ± 1.4 (*n* = 13)	22 ± 2 (*n* = 14)	20.8 ± 2.2 (*n* = 9)	22.7 ± 2 (*n* = 14)

ND, non-diabetic; STZ, streptozotocin; Los, Angiotensin II inhibitor (Losartan).

Values are mean ± s.e.m.

### Angiotensin II inhibitors accelerate wound healing in diabetic mice

**Figure 1:**
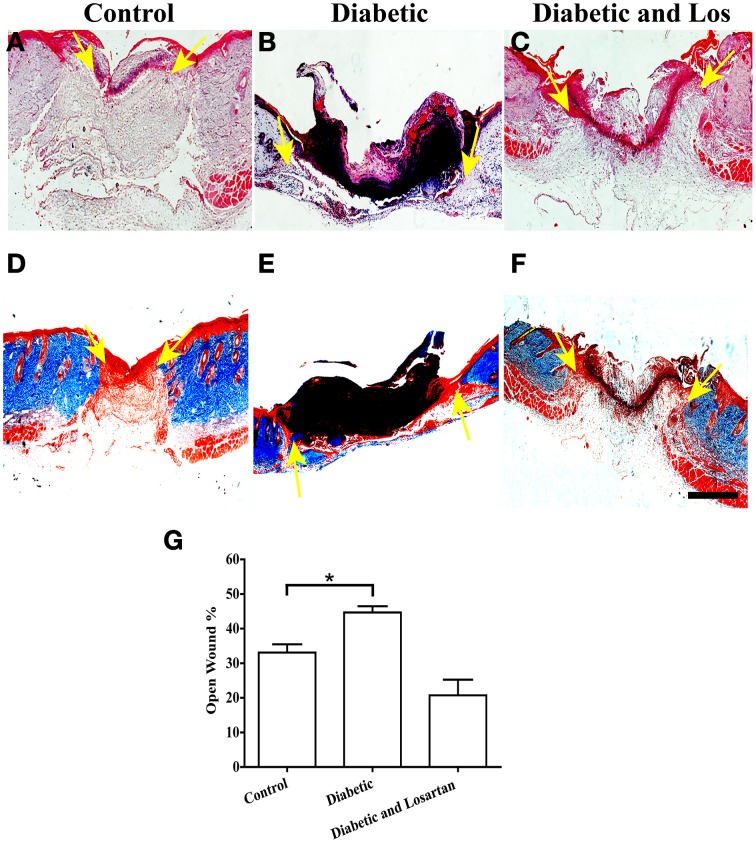
**Analysis of wounded skin following 3 days of healing process**. Treatment with angiotensin II inhibitor decreased the extensive wounded skin area observed in diabetic mice. **(A–C)** Haematoxylin and eosin staining of formalin fixed and paraffin embedded skin tissue sections from control **(A)**, diabetic **(B)**, and diabetic treated with losartan **(C)** mice. **(D–F)** Masson-trichrome staining of skin tissue sections from control **(D)**, diabetic **(E)**, and diabetic treated with losartan **(F)** mice. **(G)** Quantification of (%) wound width, which remained open after 3 days of healing. Values are mean ± s.e.m., ^*^p < 0.05, Mann-Whitney test. Arrows indicate edges of migrating epithelial fronts in open wounds. Scale bar 100 μm. Los: Losartan.

First we examined the healing process of 3-days wounds. Wounded skin sections from control, diabetic, and diabetic treated with losartan mice were subjected to qualitative and quantitative analysis. We performed hematoxylin and eosin (HE) staining to examine the tissue morphology and Masson-trichrome staining to visualize collagen fibers in the surrounding unwounded dermis. In all experimental groups, the wound healing process had been initiated and keratinocytes of the epidermis had started their migration to close the wound (Figure 1). We quantified the open wound width of 3-day wounds by measuring the distance between the gap between the leading epithelial edges and express it as % of the initial wounded area. As expected, we found that diabetic mice had significantly more open wounds compared to controls. In contrast, daily administration of the hypertensive drug, losartan, accelerated the process of wound healing and decreased the opening of the wound similarly to control group (Figure 1).

### Inhibition of angiotensin II enhance tissue remodeling of 7-days wounds in diabetic mice

**Figure 2:**
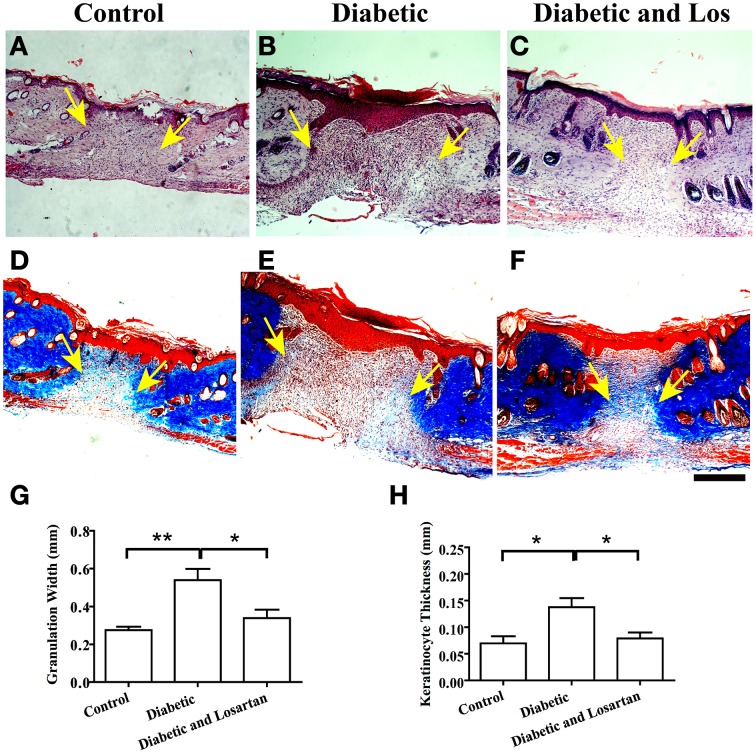
**Analysis of wounded skin following 7 days of healing process. (A–C)** Haematoxylin and eosin staining of formalin fixed and paraffin embedded skin tissue sections from control **(A)**, diabetic **(B)**, and diabetic treated with losartan **(C)** mice. **(D–F)** Masson-trichrome staining of skin tissue sections from control **(D)**, diabetic **(E)**, and diabetic treated with losartan **(F)** mice. Quantification of granulation tissue width (mm) **(G)** and the depth of newly formed keratinocyte layers (mm) **(H)** in control, diabetic and diabetic treated with Losartan mice. Both the area of the granulation tissue and the thickness of the newly formed epidermis in diabetic mice treated with losartan are similar to control and decreased compared with non-treated diabetic mice, indicating an accelerated wound healing response. Arrows indicate edges of granulation tissue area. Values are mean ± s.e.m., ^*^p < 0.05, ^**^p < 0.01, Mann-Whitney test. Arrows indicate edges of collagen fibers at the two sides of the wounded dermis. Scale bar 100 μm. Los: Losartan.

Next we performed qualitative and quantitative analysis of skin sections derived from 7-days wounds. Hematoxylin and eosin staining revealed the multi-layered epidermis formed by proliferative and migrating keratinocytes and the underlying granulation tissue (Figure 2). At this stage of wound healing, granulation tissue starts to resolve and the extracellular matrix of the dermis gets remodeled and produce large collagen fibers that can be depicted by Masson-trichrome staining (Figure 2). We observed that the diabetic mice displayed a delay in the collagen fiber formation that was reversed by administration of Losartan. We quantified the width of the wound granulation area by measuring the distance between the collagen fibers of the two sides of the wounded dermis, marking the wounded area and found that the granulation area in diabetic mice was significantly larger than the control mice, indicating impaired resolution and extracellular matrix remodeling. In contrast, the wound granulation area of diabetic mice treated with the Angiotensin II inhibitor, Losartan, was similar to control group (Figure 2).

Another indication of wound resolution is the thickness of the initially formed epidermis. Initially, keratinocytes that have proliferated and migrated to close the wound form a multi-layer undifferentiated epidermis. Subsequently, keratinocytes cease proliferation and differentiate, decreasing the thickness and altering the morphology of the initially formed epidermis. The diabetic mice displayed increased thickness of the newly assembled epidermis compared to control group (Figure 2). Consistent with Losartan stimulating the wound healing response, the depth of epidermal layers in diabetic mice treated with the angiotensin II inhibitor, was similar to control group and thinner than diabetic mice (Figure 2). Taken these data together, Losartan facilitated the remodeling of 7-days wounds in diabetic mice and displayed a comparable to control wound morphology.

### No adverse effect of angiotensin II inhibitors in healed wounds

**Figure 3:**
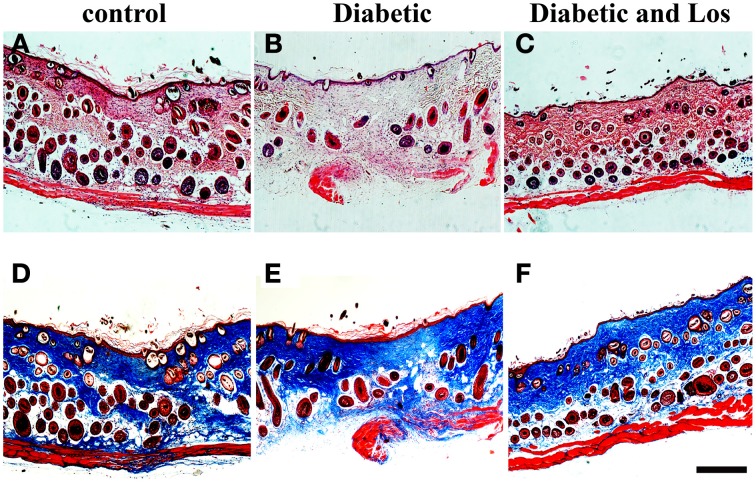
**Analysis of wounded skin following 14 days of healing process**. No differences were observed among the different experimental groups at 14 days post-wounded. Closed wounds exhibited collagen staining and formation of new hair follicles. **(A–C)** Haematoxylin and eosin staining of formalin fixed and paraffin embedded skin tissue sections from control **(A)**, diabetic **(B)**, and diabetic treated with losartan **(C)** mice. **(D–F)** Masson-trichrome staining of skin tissue sections from control **(D)**, diabetic **(E)** and diabetic treated with losartan **(F)** mice. Scale bar 100 μm. Los: Losartan.

Lastly, we examined skin sections of fully closed and resolved wounds. As expected, after 14 days of wound healing, the skin epidermis was fully differentiated and new hair follicles were developed in all experimental groups (Figure 3). The granulation tissue was fully resolved and a collagen-rich matrix assembled in skin dermis of all experimental mice (Figure 3). We did not observe any morphological differences in the skin of diabetic mice treated with Angiotensin II inhibitor compared to control group, indicating that Losartan does not affect normal skin.

### Angiotensin II inhibitors normalise wound stroma

**Figure 4:**
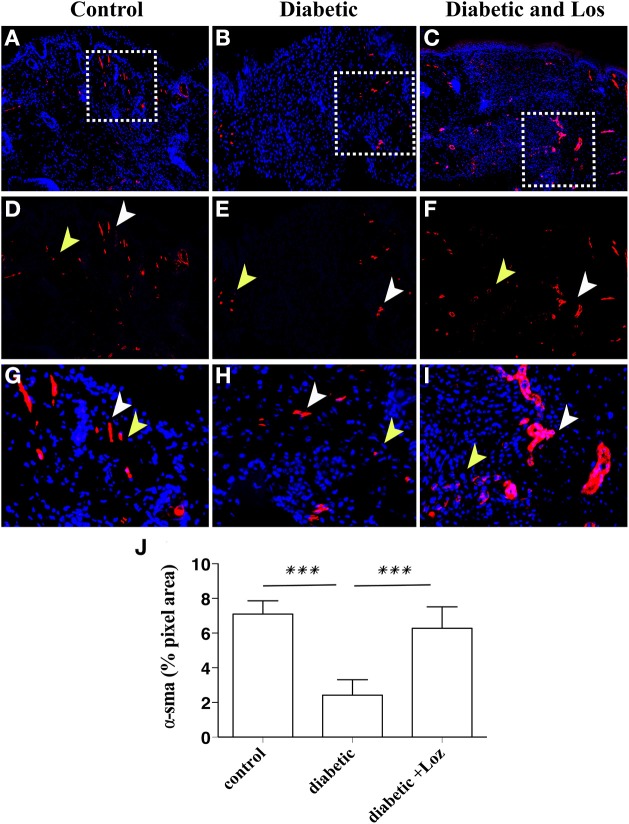
**Immunofluorescence analysis of α-SMA expression on wounded murine skin tissue sections**. Formalin fixed paraffin embedded tissue was stained for α-SMA (red). Cell nuclei were stained with DAPI (blue). Examples are shown from control **(A)**, diabetic **(B)**, and diabetic treated with losartan **(C)** mice. The granulation tissue area in diabetic mice treated with Losartan **(C,I)** display similar to control **(A,G)** and increased compared with diabetic non-treated mice **(B,H)**, number of a-SMA positive cells. **(D–I)** Magnification of the areas marked in (**A–C**) with the white boxes. **(J)** Quantification of a-SMA immunofluorescence (pixel area, arbitrary units). White arrowheads indicate blood vessels and yellow arrowheads indicate myofibroblasts. Values are mean ± s.e.m., ^***^p < 0.001, Mann-Whitney test.

To gain insight into the mechanism of Losartan function in accelerating wound healing in diabetic mice, we investigated further the wound healing process. Intrigued by the reduced wound opening and the decreased granulation area observed in wounds of diabetic mice treated with Losartan, we hypothesize that inhibiting Angiotensin II could alter the contraction capacity of the wound. Thus, we examined the presence of myofibroblasts, the cell constituent of wound stroma that is responsible for tissue contraction (Li and Wang, 2011), by staining wound sections with α-smooth muscle actin (α-SMA). The wounds of diabetic mice seem to have lower levels of α-SMA expression, compared to the other groups (Figure 4). Treatment with Losartan reverted this phenotype. The presence of α-SMA positive cells in wounds of diabetic mice treated with Losartan was similar to control group (Figure 4).

**Figure 5:**
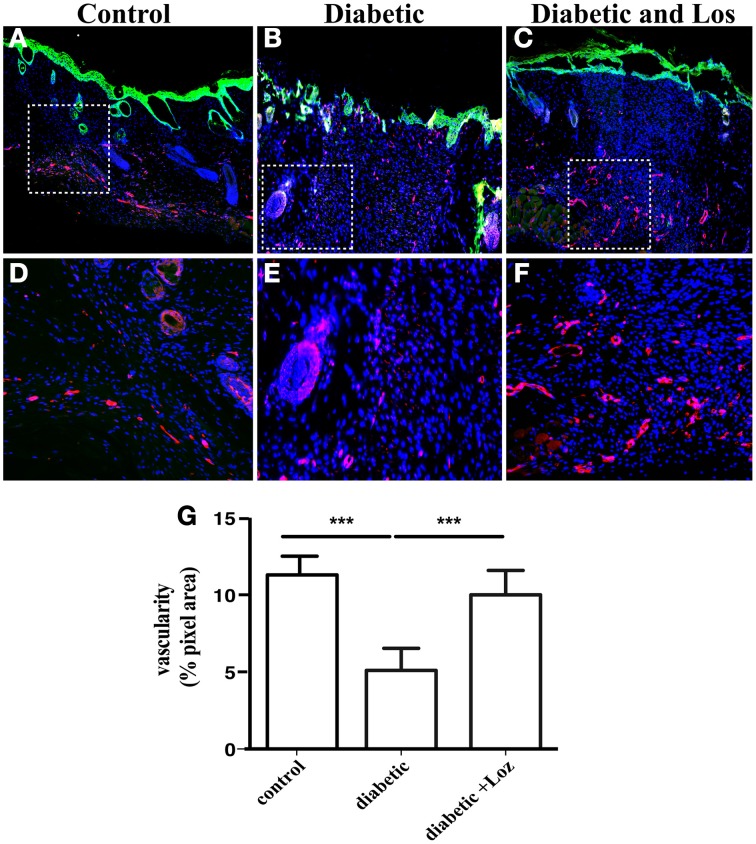
**Immunofluorescence analysis of newly formed epidermis and skin blood vessels**. Formalin fixed paraffin embedded tissue sections were stained for undifferentiated epidermal cells by keratin-14 (green) and vascular endothelium by endomucin (red) from control **(A)**, diabetic **(B)**, and diabetic treated with losartan **(C)** mice. Cell nuclei were stained with DAPI (blue). The granulation tissue of diabetic mice treated with losartan **(C,F)** seems to show a higher degree of vascularization when compared to diabetic mice **(B,E)** or control **(A,D)**. **(D–F)** Magnification of the areas marked in (**A–C**) with the white boxes. **(G)** Quantification of endomucin immunofluorescence (pixel area, arbitrary units). Values are mean ± s.e.m., ^***^p < 0.001, Mann-Whitney test.

Expression of α-SMA is also present in pericytes and smooth muscle cells surrounding blood vessels. Altered permeability of existing vasculature and the formation of new blood vessel are required for tissue regeneration and might explain the differences in the wound healing of diabetic mice elicited by Losartan treatment. The analysis of wound sections stained with α-SMA revealed increased number of larger structures that resemble blood vessels. Therefore, we sought to analyse differences in the presence of blood vessels in wound stroma. We examined wounds of mice that were still open or recently bridged together as indicated by a thin layer of undifferentiated keratinocytes, stained by keratin-14 (Figure 5), as these wounds have active angiogenesis (Eming et al., 2007). Staining of wound sections with endomucin, a marker of vascular endothelial cells revealed that wound stroma of diabetic mice treated with Losartan was rich in blood vessel, similar to control group (Figure 5). Consistent with the impaired vascular function that is associated with diabetes, wound stroma from diabetic mice had fewer blood vessels compared to control or diabetic treated with Lozartan group (Figure 5). Taken together, these findings indicate that Losartan could affect wound contraction and blood vessel function, acting to normalize the wound stroma and facilitate wound healing in diabetic mice.

## Discussion

Tissue regeneration is severely impaired in diabetes, as exemplified by chronic wounds and ulcers and microvasculature aberrations. In diabetic skin tissues the renin angiotensin system is activated with increased expression of angiotensin II and elevated expression ratio between the type 1 (AT1) and type 2 (AT2) receptors (Hao et al., 2011). The purpose of the current study was to elucidate whether the treatment with the angiotensin II inhibitor, Losartan (an anti-hypertensive drug) affects tissue regeneration in diabetes. For this we examined the wound healing process of the skin epidermis in streptozotocin-induced diabetic mice. We found that treatment with Losartan for the duration of the wound healing process accelerated tissue regeneration and reversed the morphological alterations induced by diabetes.

We didn't observe any difference in wound healing responses between non-diabetic and non-diabetic mice treated with Losartan. However, complete depletion of the AT1 receptor delayed the wound healing in the skin of mice (Kawaguchi et al., 2004; Yahata et al., 2006). One possible explanation of this discrepancy could be that, in contrast to an acute inhibition of the AT1 receptor through the administration of a selective drug, chronic absence of AT1 action might have generated compensatory signals from AT2 receptor or the angiotensin-converting enzyme (ACE), which could differentially influence wound healing responses. It is well established that the final outcome of Angiotensin II function is tightly controlled by the action of AT1 and AT2 receptors, which often exhibit opposing effects and the angiotensin-converting enzyme (ACE) (Tamarat et al., 2002; Takeda et al., 2004; Yahata et al., 2006). Based on our data showing that angiotensin II inhibition facilitates the wound healing under diabetic conditions, it would be interested to examine the effect of AT1 deletion in diabetes.

Several studies have elucidated the main defects of wound healing in diabetes (Werner et al., 1994; Loots et al., 1998). This disease is mainly characterized by severe vascular abnormalities as a result of blood vessel dysfunction that can directly influence the wound healing process, both on the initial anti-inflammatory stage and on the re-epithelisation phase and reconstitution of the injured dermis, leading to important delays on the onset of the reaction and dysfunction in keratinocyte migration, extracellular matrix remodeling, and resolution of the granulation tissue. Consistent with this, all phases of the wound healing process were impaired in streptozotocin-induced diabetic animals. The re-epithelisation was significantly delayed at 3-days of wound healing and both the area of granulation tissue and the thickness of the newly formed undifferentiated epidermis were increased at 7-days wounds. Treatment with Losartan for the duration of the wound healing process was sufficient to reverse these morphological differences and to induce a normal-like wound healing response in diabetic mice. The decreased granulation area and the restoration of collagen production in the wounds of diabetic mice treated with Losartan indicate reduced fibrosis and sufficient remodeling of extracellular matrix. Our data agree with studies showing that Losartan can decrease profibrotic signals by inhibiting thrombospondin -1 and transforming growth factor (TGF)-β1 action and downregulating connective tissue growth factor (CCN2) (Chauhan et al., 2013). Additionally, treatment of primary fibroblasts isolated from diabetic skin with Losartan downregulated the TGF-β1 and TIMP-1 expression that seem to be involved in the impaired collagen formation of the diabetic skin (Ren et al., 2013).

Our findings suggest that inhibition of angiotensin II function in diabetes could normalize skin responses and facilitate tissue regeneration. We observed that Losartan treatment of diabetic mice resulted in an enrichment of sma-expressing myofibroblasts and a higher degree of vascularization in the wound stroma, facilitating wound contraction and normalizing wound healing responses. The decreased number of sma-positive cells detected in the wounds of diabetic mice could contribute to the impaired wound healing. It is well established that myofibroblasts have essential roles in wound healing, secreting growth factors that facilitate the proliferation and migration of epithelial cells, and mediating the mechanical contraction of the wound (Werner and Grose, 2003; Li and Wang, 2011; Eming et al., 2014). Consistent with this, a recent study showed that the presence of a-SMA positive peripheral blood cells and myofibroblasts accelerates the wound repair process in a diabetic mouse model, mainly by promoting cell proliferation, re-epithelialization, and angiogenesis (Kao et al., 2011). Conflicting data exists regarding the effect of AT1 inhibitors in angiogenesis. It has been reported that angiotensin II signaling through AT1 receptor increase vascular endothelial growth factor (VEGF) expression and facilitates vascular permeability and angiogenesis (Fujita, 2004; Suganuma et al., 2005). In contrast to the above studies, Losartan treatment was shown to improve vascular perfusion and decompress blood vessels in tumors, without affecting VEGF expression (Diop-Frimpong et al., 2011; Chauhan et al., 2013). Our data are consistent with a vascular normalization effect of angiotensin II inhibitor, which promotes wound healing in diabetes. There is evidence that a normalized and a healthy peripheral circulation in diabetic mice improve cutaneous wound healing (Pietramaggiori et al., 2009). Also, a recent study showed that pretreatment of streptozotocin-induced diabetic mice with a mixture of pro-angiogenic growth factors and endothelial progenitor cells (EPCs) accelerated incisional wound healing (Ackermann et al., 2014), indicating that restoring a functional vasculature in diabetes could improve the wound repair process and facilitate tissue regeneration.

Taken together, our results revealed a beneficial role of losartan in accelerating cutaneous wound healing in a streptozotocin-induced diabetic mouse model, by restoring wound stroma responses through its action on blood vessels and myofibroblasts. Further studies will elucidate the precise role of this antihypertensive drug in facilitating tissue regeneration of different organs in diabetic conditions. Given that Losartan is a safe drug, already used in clinical practice to treat hypertension, our results highlight a novel therapeutic approach that could have a possibly direct clinical impact on improving cutaneous wound therapy for diabetic patients.

### Conflict of interest statement

The authors declare that the research was conducted in the absence of any commercial or financial relationships that could be construed as a potential conflict of interest.

## Supplementary material

Supplementary Figure 1 **Losartan has no effect on the wound healing of non diabetic mice. (A)** Quantification of (%) wound width, which remained open after 3 days of healing, as in Figure 1. **(B)** Quantification of granulation tissue width (mm) in 7-days wounds, as in Figure 2. Values are mean ± s.e.m.
