# Supplementary figures and images for: Angiotensin II inhibitor facilitates epidermal wound regeneration in diabetic mice
Source: Front Physiol. 2015 Jun 9;6:170. doi: 10.3389/fphys.2015.00170 (PMC4460301; doi:10.3389/fphys.2015.00170)

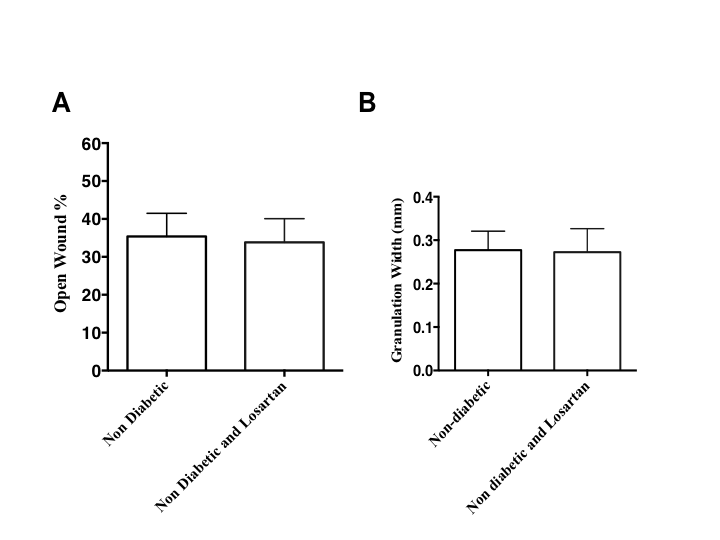

Supplement: Supplementary Figure 1 — Losartan has no effect on the wound healing of non diabetic mice. (A) Quantification of (%) wound width, which remained open after 3 days of healing, as in Figure 1. (B) Quantification of granulation tissue width (mm) in 7-days wounds, as in Figure 2. Values are mean ± s.e.m. [file Image1.TIFF]
